# Phylogeographic Analysis and Genetic Structure of an Endemic Sino-Japanese Disjunctive Genus *Diabelia* (Caprifoliaceae)

**DOI:** 10.3389/fpls.2019.00913

**Published:** 2019-07-16

**Authors:** Kun-Kun Zhao, Sven Landrein, Russell L. Barrett, Shota Sakaguchi, Masayuki Maki, Wei-Xue Mu, Ting Yang, Zhi-Xin Zhu, Huan Liu, Hua-Feng Wang

**Affiliations:** ^1^Hainan Key Laboratory for Sustainable Utilization of Tropical Bioresources, School of Life and Pharmaceutical Sciences, Hainan University, Haikou, China; ^2^Xishuangbanna Tropical Botanical Garden, Chinese Academy of Sciences, Mengla, China; ^3^National Herbarium of New South Wales, Royal Botanic Gardens and Domain Trust, Sydney, NSW, Australia; ^4^Graduate School of Human and Environmental Studies, Kyoto University, Kyoto, Japan; ^5^Botanical Gardens, Tohoku University, Sendai, Japan; ^6^BGI-Shenzhen, Beishan Industrial Zone, Shenzhen, China; ^7^State Key Laboratory of Agricultural Genomics, BGI-Shenzhen, Shenzhen, China

**Keywords:** *Diabelia*, Caprifoliaceae, phylogeography, ecological niche modeling, Sino-Japanese disjunct distribution

## Abstract

The Sino-Japanese Floristic Region (SJFR) is a key area for plant phylogeographical research, due to its very high species diversity and disjunct distributions of a large number of species and genera. At present, the root cause and temporal origin of the discontinuous distribution of many plants in the Sino-Japanese flora are still unclear. *Diabelia* (Caprifoliaceae; Linnaeoideae) is a genus endemic to Asia, mostly in Japan, but two recent discoveries in China raised questions over the role of the East China Sea (ECS) in these species' disjunctions. Chloroplast DNA sequence data were generated from 402 population samples for two regions (*rpl*32-*trn*L, and *trn*H-*psb*A) and 11 nuclear microsatellite loci were screened for 549 individuals. Haplotype, population-level structure, combined analyses of ecological niche modeling, and reconstruction of ancestral state in phylogenies were also performed. During the Last Glacial Maximum (LGM) period after the Tertiary, *Diabelia* was potentially widely distributed in southeastern China, the continental shelf of the East China Sea and Japan (excluding Hokkaido). After LGM, all populations in China have disappeared except those in Zhejiang which may represent a Glacial refuge. Populations of *Diabelia* in Japan have not experienced significant bottleneck effects, and populations have maintained a relatively stable state. The observed discontinuous distribution of *Diabelia* species between China and Japan are interpreted as the result of relatively ancient divergence. The phylogenetic tree of chloroplast fragments shows the characteristics of multi-origin evolution (except for *D. sanguinea*). STRUCTURE analysis of nuclear Simple Sequence Repeat (nSSR) showed that the plants of the *Diabelia* were divided into five gene pools: *D. serrata, D. spathulata, D. sanguinea, D. ionostachya* (*D. spathulata* var. *spathulata*-Korea), and populations of *D. ionostachya* var. *ionostachya* in Yamagata prefecture, northern Japan. Molecular evidence provides new insights of *Diabelia* into biogeography, a potential glacial refuge, and population-level genetic structure within species. In the process of species differentiation, ECS acts as a corridor for two-way migration of animals and plants between China and Japan during glacial maxima, providing the possibility of secondary contact for discontinuously distributed species between China and Japan, or as a filter (creating isolation) during glacial minima. The influence of the ECS in speciation and biogeography of *Diabelia* in the Tertiary remains unresolved in this study. Understanding origins, evolutionary histories, and speciation will provide a framework for the conservation and cultivation of *Diabelia*.

## Introduction

The northern hemisphere in the early Tertiary period was the source of significant speciation events at medium to high latitudes due to its warm and humid climate (Tiffney, 1985a,b). Beginning in the early Pleistocene, these species began to migrate gradually to low latitudes as the climate became colder (Wolfe, 1971). From the late tertiary to the beginning of the Quaternary, three Glacial (Ice Age) refuge areas developed in East Asia, North America, and southwestern Europe. In East Asia, the only limited glacial cover was formed during the Quaternary glacial period (Qian, 1999). Coupled with a complex geographical environment, a very high number of isolated phylogenetic lineages survived in East Asia (Wen et al., 1998; Wen, 1999; Qiu et al., 2011). The Sino-Japanese flora (SJFR) in East Asia has the largest diversity of temperate plant species in the world (Qiu et al., 2011), and is also a very significant glacial refuge during the Quaternary Ice Age, with significant population fragmentation and subsequent allopatric speciation (Qiu et al., 2011). The Land-bridge islands, formed by the China, continental shelf of the East China Sea and the Japanese archipelago (Whittaker et al., 2007), is an ideal environment for studying the genetic effects of geographic isolation on species formation (Li et al., 2010; Qiu et al., 2011). It differs from two neighboring floristic regions, the Qinghai Tibetan Plateau and the Sino Himalayan regions as its fragmentation is dominated by sea level changes in the East China (Qian and Ricklefs, 2000; Qiu et al., 2011). Palaeobiome reconstructions have demonstrated that the warm deciduous forests of the SJFR extended over vast regions of the mainland (c. 1 million km^2^) that developed in the East China Sea (ECS) during the Last Glacial Maximum (LGM; c. 21,000 years BP) as well as cool Quaternary periods when ocean levels were c. 85–140 m lower (Millien-Parra and Jaeger, 1999; Wang, 1999; Siddall et al., 2003). Many uncertainties remain about the ECS's role in speciation processes in the SJFR (Qiu et al., 2011; Zhai and Silman, 2012). Some studies have shown that SJFR provides a strong biogeographic barrier, with *Kalopanax semptemlobus* exhibiting strong genetic differentiation or even species differentiation on both sides of the ECS Qiu et al., 2009a; Sakaguchi et al., 2012. However, yet another study shows no obvious genetic differentiation (e.g., *Cercidiphyllum japonicum*; Qi et al., 2012). Hence, whether ECS affects the differentiation of discontinuously distributed plants on both sides of the East China Sea remains unclear, so it is necessary to explore the role of the ECS in speciation and species migration through additional case studies. Few studies have examined the very uneven distribution of species diversity between China and Japan, with most studies focusing on species that are widely distributed in Japan (Sakaguchi et al., 2012). Moreover, the most detailed studies only focused on a single species, and few studies have included all the species of a genus or a monophyletic clade (Zhai and Silman, 2012).

**Figure 1:**
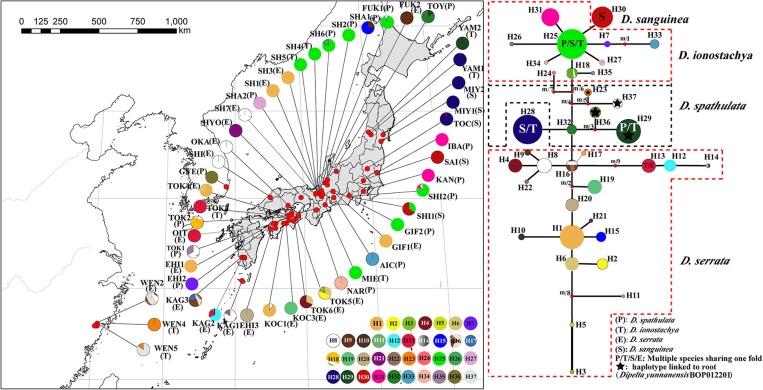
Species range, geographical distribution of haplotypes, and phylogenetic relationship among four *Diabelia* species. Geographical distribution of 37 chloroplast haplotypes in sampled locations. Shading indicates the native range of each species (Hara, 1983; Landrein and Farjon, 2019). Four haplotypes (H13, H25, H28, and H29) are multiple species shared haplotypes, of which H25 is shared haplotype of *D. ionostachya, D. sanguinea*, and *D. spathulata*. H13 is the shared haplotype for *D. ionostachya* and *D. serrata*; H28 is the shared haplotype for *D. ionostachya* and *D. sanguinea*; H29 is the shared haplotype for *D. ionostachya* and *D. spathulata*, respectively.

*Diabelia* is a genus in Caprifoliaceae subfamily Linnaeoideae that was recently segregated from *Abelia* (Landrein, 2010; Wang et al., 2015). *Diabelia* has been resolved as sister to the genus *Dipelta*, and together they form a clade with *Kolkwitzia* (Wang et al., 2015). While the genus is very distinct, species delimitation within *Diabelia* is controversial and unclear at present. Traditionally, *Diabelia* included three species with an unusually disjunct distribution pattern in Japan, East China, and Korea (Liang et al., 2004; Zhou and Wen, 2006; Yang et al., 2011; Shin et al., 2012; see Figure 1). Hara (1983) recognized three species primarily based on the number and length of sepals, with additional varieties and forms recognized on the basis of hair types, nectary shape, fusion of nectaries and corolla, and corolla color. *Diabelia spathulata* (Sieb. & Zucc.) Landrein is distributed in South, central and northern areas of Japan (from Aomori to Saga Prefectures) except Hokaido (Hara, 1983) and three varieties recognized (as *Abelia spathulata* var. *spathulata*, var. *sanguinea* Makino, and var. *stenophylla* Honda). The second species, *D. serrata* (Sieb. & Zucc.) Landrein, is widely distributed in southern Japan (from Shiga to Kagoshima Prefectures) and also contains a number of infra-specific varieties and forms (as *Abelia serrata* var. *serrata* f. *serrata*, var. s*errata* f. *obspathulata* (Koidz.) Sugimoto, and f. *tomentosa* (Koidz.) Nakai). These two species also have scattered distributions along the southeast coast of East China (e.g., Sihaishan Mts, Wenzhou, Zhejiang; Liang et al., 2004; Zhou and Wen, 2006). The third taxon, *D. tetrasepala* (Koidz.) Landrein, is a narrow endemic from Japan (from Fukushima to Fukuoka Prefectures), where its range partly overlaps with both *D. spathulata* and *D. serrata* (Hara, 1983). The number of sepals is generally regarded as a key feature to differentiate *Diabelia* species, i.e., there are five sepals in *D. spathulata*, two or three sepals in *D. serrata* and four large plus a reduced sepal in *D. ionostachya* var. *tetrasepala* (Koidz.) Landrein (Hara, 1983; Landrein, 2010; Landrein and Farjon, 2019). However, based on recent herbarium studies and field work, we found that the number of sepals is not stable in *Diabelia*, rather the number of sepals ranges from two to five in *D. serrata*, from four to five in *D. spathulata* and there are four large and one reduced sepal in *D. ionostachya* var. *tetrasepala*. Landrein and Farjon (2019) have proposed the recognition of four species (i.e., *D. serrata, D. spathulata, D. sanguinea*, and *D. ionostachya* (Nakai) Landrein) based on morphological characters (the number of sepals, nectary cushion position and corolla color). *Diabelia serrata* is distributed in Shikoku, Kyushu and the central and southern regions of Honshu. Whereas, *Diabelia ionostachya* is mainly distributed in Honshu and Shikoku, but rarely in Kyushu. *Diabelia sanguinea* grows mainly in central and northeastern Honshu. *Diabelia spathulata* is widely distributed in Honshu and Shikoku, but rare in Kyushu and Korea. A small number of populations of *D. serrata* and *D. ionostachya* are distributed in Zhejiang, China Landrein and Farjon, 2019. This revision maintains the definition of two species recognized by Hara (1983), *D. serrata* and *D. spathulata*. *Abelia spathulata* var. *sanguinea* is recognized at species level as *D. sanguinea* (Makino) Landrein. The overlooked name *Abelia ionostachya* Nakai is recombined in *Diabelia* and two varieties are recognized, var. *stenophylla* Landrein and var. *tetrasepala*. For convenience and uniformity, the samples used in our analyses were assigned names based on Landrein and Farjon's proposed morphology-based taxonomy.

The small fruits (achenes) of *Diabelia* are likely to be wind dispersed (Zhou and Wen, 2006) and might be easily blown across the ECS by strong winds associated with typhoons or large storms. The sister lineage of *Diabelia* is *Dipelta*, which is endemic to southwest China (e.g., Shaanxi, Gansu, and Yunnan province), and while *Diabelia* is mainly distributed in Japan, its origin and subsequent dispersal patterns remain uncertain (Wang et al., 2015).

In the present investigation, we assessed the phylogenetic relationships and the phylogeographic history of *Diabelia* taxa to determine when these species diverged, and which processes (dispersal or vicariance) most likely led to the current disjunct distribution of *D. ionostachya* and *D. serrata* across the ECS. We utilized population-level sampling to determine the relations between species and potential hybrid populations in *Diabelia*. Our objectives were (i) to refine the timeframe in which *Diabelia* populations diverged by fitting both datasets (nSSR and cpDNA) to an Isolation with Migration (IM) model; (ii) to model the species' potential distributions in response to past climatic changes (LGM) and to the present day using Ecological Niche Modeling (ENM); (iii) Through a comprehensive analysis of nSSR and cpDNA data we aimed to determine whether the current biogeography of *Diabelia* is due to the spread from China or Japan by long-distance dispersal (e.g., typhoons) or during the LGM. Finally, we questioned whether *D. ionostachya* var. *tetrasepala* could be a hybrid of *D. serrata* (calyx with 2–3 sepals) and *D. spathulata* or *D. ionostachya* (calyx with five sepals). We used these data to assess infra-specific variation within *Diabelia* species.

## Materials and Methods

### Plant Material and Sampling Design

We collected leaf samples from 602 *Diabelia* individuals from 69 sites throughout the range of this genus in Japan (64 populations), East China (Zhejiang, four populations) and Korea (one population, Figure 1; Table 1). Chloroplast (cp) DNA data were generated from 402 samples (representing all 69 sites) and nSSR data was generated from 549 individuals (Table 2). Samples were selected to provide a representation of both geographic and morphological diversity within the genus. As some specimens were not fertile at the time of collection, or their identity was not recorded previously, morphological data for key reproductive organ traits could not be assessed.

**Table 1:** Details of sampled populations of *Diabelia* included in this study.

**Number**	**Code**	**Taxon**	**General collection site**	**Latitude (^**°**^N)**	**Longitude (^**°**^E)**	**Altitude (m)**
1	WEN	*Diabelia serrata*	Sihaishan, Yongjia, Wenzhou, Zhejiang, China	28.511	120.729	931
2	KAG1	*Diabelia serrata*	Mt. Hizukushi, Yakushima-cho, Kumage-gun, Kagoshima, Japan	30.330	130.397	410
3	HYO	*Diabelia serrata*	Hirone, Inagawa-cho, Kawabe-gun, Hyogo, Japan	34.898	135.331	205
4	SHI	*Diabelia serrata*	Kakiki-mura, Kanoashi-gun, Shimane, Japan	34.308	131.825	814
5	EHI1	*Diabelia serrata*	Mt. Ishiduchi, Ehime, Japan	33.770	133.183	1,414
6	SH1	*Diabelia serrata*	Tanakamisato-cho, Otsu city, Shiga, Japan	34.917	135.978	501
7	SH3	*Diabelia serrata*	Fujise, Taga-cho, Inukami-gun, Shiga, Japan	35.176	136.297	239
8	SH7	*Diabelia serrata*	Kutsukake, Nishiasai-cho, Nagahama city, Shiga, Japan	35.580	136.114	329
9	SHA1	*Diabelia spathulata* var. *spathulata*	Kowaki-cho, Higashiomi city, Shiga, Japan	35.124	136.183	324
10	FUK2	*Diabelia serrata*	Mt. Aoba, Takahama-cho, Ooi-gun, Fukui, Japan	35.494	135.490	110
11	TOK4	*Diabelia serrata*	Mt. Takamaru, Kamikatsu, Tokushima, Japan	33.888	134.326	1,380
12	GIF1	*Diabelia serrata*	Takaga, Seki city, Gifu, Japan	35.643	136.843	300
13	TOK5	*Diabelia serrata*	Mt. Shozanji, Kamiyama, Tokushima, Japan	33.982	134.306	880
14	TOK6	*Diabelia serrata*	Mt. Koutsu, Yoshinogawa city, Tokushima, Japan	34.017	134.196	1,100
15	OKA	*Diabelia serrata*	Go-Kei, Okayama, Japan	34.705	133.721	26
16	KAG2	*Diabelia serrata*	Byakushi ike pond, Kagoshima, Japan	31.954	130.838	1,316
17	OIT	*Diabelia serrata*	Mt. Hareyama, Oita, Japan	33.242	131.140	750
18	KAG3	*Diabelia serrata*	Takakuma, Kagoshima, Japan	31.320	130.84	163
19	KOC3	*Diabelia serrata*	Mt. Shiraga, Motoyama-cho, Kochi, Japan	33.815	133.592	1,363
20	KOC1	*Diabelia serrata* f. *buchwaldii*	Takanose-kyo gorge, Kito, Naka-cho, Kochi, Japan	33.760	133.762	1,224
21	EHI3	*Diabelia serrata* f. *buchwaldii*	Mt. Higashiakaishi, Ehime, Japan	33.875	133.373	1,688
22	NAG	*Diabelia serrata*	Nagasaki, Japan	32.740	130.258	900
23	WEN2	*Diabelia serrata*	Yongjia Sihaishan, Wenzhou, Zhejiang, China	28.519	120.738	670
24	YMS1	*Diabelia serrata*	Kofu-shi city, Yamanashi, Japan	35.710	138.58	778
25	TOK7	*Diabelia serrata*	Ogawara plateau,Tokushima, Japan	33.964	134.427	633
26	YAC	*Diabelia serrata*	Yamaguchi, Japan	34.314	131.576	240
27	KOC4	*Diabelia serrata* f. *tomentosa*	Shimanto Town, Kochi Prefecture, Shikoku Island, Japan	33.290	132.880	114
28	YAM1	*Diabelia ionostachya* var. *ionostachya*	Hatasawa, Obanasawa city, Yamagata, Japan	38.546	140.442	230
29	YAM2	*Diabelia ionostachya* var. *ionostachya*	Hataya, Yamanobe-machi, Higashi-Murayama-gun, Yamagata, Japan	38.238	140.187	660
30	TOK3	*Diabelia ionostachya var. tetrasepala*	Mt. Takamaru, Kamikatsu, Tokushima, Japan	33.886	134.328	1,210
31	YMS2	*Diabelia spathulata* var. unknown	Hirasemachi, Kofu-shi city, Yamanashi, Japan	35.710	138.54	928
32	SHI2	*Diabelia spathulata* var. *spathulata*	Izu city, Banjiro, Shizuoka, Japan	34.860	139.021	1,300
33	SH4	*Diabelia ionostachya* var*. tetrasepala*	Fujise, Taga-cho, Inukami-gun, Shiga, Japan	35.176	136.297	239
34	MIE	*Diabelia ionostachya* var*. tetrasepala*	Taian-cho, Inabe city, Mie, Japan	35.108	136.442	689
35	SH2	*Diabelia spathulata* var*. spathulata*	Omori-cho, Higashiomi city, Shiga, Japan	35.078	136.231	158
36	SH5	*Diabelia ionostachya* var*. tetrasepala*	Tominoo, Taga-cho, Inukami-gun, Shiga, Japan	35.195	136.301	171
37	SH6	*Diabelia spathulata* var*. spathulata*	Kutsukake, Nishiasai-cho, Nagahama city, Shiga, Japan	35.580	136.114	329
38	SHA2	*Diabelia spathulata* var. unknown	Kowaki-cho, Higashiomi city, Shiga, Japan	35.124	136.183	324
39	EHI2	*Diabelia spathulata* var*. spathulata*	Kawanouchi, Toon city, Ehime, Japan	33.751	132.982	760
40	SHI1	*Diabelia sanguinea*	Mt. Kinkan, Numadzu city, Shizuoka, Japan	34.975	138.834	813
41	KOC2	*Diabelia spathulata*	Takanose-kyo gorge, Kito, Naka-cho, Kochi, Japan	33.760	133.762	1,224
42	KAN	*Diabelia spathulata* var*. spathulata*	Mt. Ishizare, Kanagawa, Japan	35.580	139.174	375
43	IBA	*Diabelia spathulata* var*. spathulata*	Mt. Tsukuba, Ibaraki, Japan	36.234	140.120	390
44	FUK1	*Diabelia spathulata* var*. spathulata*	Kami-yoshino, Eiheiji-cho, Yoshida-gun, Fukui, Japan	36.049	136.311	415
45	AIC	*Diabelia spathulata* var*. spathulata*	Kami-sanbonmatsu, Honzyuku-cho, Okazaki city, Aichi, Japan	34.897	137.263	105
46	NAR	*Diabelia spathulata* var*. spathulata*	Shirakawa, Kita-Kamiyama-mura, Yoshino-gun, Nara, Japan	34.108	135.991	350
47	TOK1	*Diabelia spathulata* var*. spathulata*	Mt. Shozanji, Kamiyama, Tokushima, Japan	33.982	134.305	900
48	TOK2	*Diabelia spathulata* var*. spathulata*	Mt. Takamaru, Kamikatsu, Tokushima, Japan	33.883	134.331	1,090
49	GIF2	*Diabelia spathulata* var*. spathulata*	Zyourinji, Toki city, Gifu, Japan	35.387	137.196	270
50	WEN4	*Diabelia ionostachya* var. *wenzhouensis*	Yongjia Sihaishan, Wenzhou, Zhejiang, China	28.519	120.738	670
51	WEN5	*Diabelia ionostachya* var. *wenzhouensis*	lushanzhulin, Sihanshan, Wenzhou, Zhejiang, China	28.695	120.708	590.9
52	NII	*Diabelia spathulata*	Kashiwazaki, Niigata, Japan	37.330	138.609	55
53	TOK8	*Diabelia serrata*	Takanose-kyo gorge, Kito, Naka-cho, Naka, Tokushima, Japan	33.790	134.080	612
54	GYE	*Diabelia spathulata* var. *spathulata*	Naewon temple forests,Yangsan-si, Gyeongsangnam-do, Korea	35.423	129.118	400
55	MIY2	*Diabelia sanguinea*	Shichigzyuku, town, Miyagi, Japan	37.955	140.511	471
56		*Diabelia sanguinea*	Miyato Island, Higashi-Matsushima-cho, Miyagi, Japan	38.343	141.168	5
57	TOC	*Diabelia sanguinea*	Yumoto, Nasu-cho, Nasu-gun, Tochigi, Japan	37.118	140.008	1,040
58	SAI	*Diabelia sanguinea*	Ryogami-susuki, Ogano-machi, Chichibu-gun, Saitama, Japan	36.007	138.968	390
59	TOY	*Diabelia spathulata*	Zike, Toyama city, Toyama, Japan	36.559	137.223	220
60	WEN6	*Diabelia ionostachya* var*. wenzhouensis*	Sihaishan Yongjia, Wenzhou, Zhejiang, China	28.519	120.738	994
61	FUK3	*Diabelia ionostachya* var*. ionostachya*	Nisiaizu-machi, Yama-gun, Fukushima Pref, Japan	37.573	139.584	330
62	FUK4	*Diabelia spathulata* var*. spathulata*	Taira, Iwaki-shi, Fukushima Pref, Japan	37.086	140.892	70
63	FUK5	*Diabelia ionostachya* var*. tetrasepala*	Taira, Iwaki-shi, Fukushima Pref, Japan	37.086	140.892	70
64	FUK6	*Diabelia sanguinea*	Kanayagawa, Fukushima-shi, Fukushima Pref, Japan	37.684	140.453	180
65	FUK7	*Diabelia sanguinea*	Kanayagawa, Fukushima-shi, Fukushima Pref, Japan	37.684	140.453	180
66	FUK8	*Diabelia sanguinea*	Nishishinden, Nihonmatsu-shi, Fukushima Pref, Japan	37.533	140.549	330
67	FUK9	*Diabelia spathulata* var*. spathulata*	Taira, Iwaki-shi, Fukushima Pref, Japan	37.040	140.941	60
68	FUK10	*Diabelia ionostachya* var*. tetrasepala*	Tabitomachi, Iwaki-shi, Fukushima Pref, Japan	36.929	140.634	369
69	TNS	*Diabelia ionostachya* var*. tetrasepala*	Tsukuba Botanical Garden, Japan	36.101	140.111	25.6

**Table 2:** Genetic characteristics of 59 *Diabelia* populations from China, Japan, and Korean surveyed for chloroplast (cp) DNA sequences and nSSR variation.

**Code**	**Species name**	**Microsatellites**									**cpDNA**			
		**N1**	**P (99%)**	**A**	**TA**	**PA**	**RA**	**Ho**	**He**	**Fis**	**N2**	**cp–Hd**	**cp—π (×10^**−2**^)**	**Hap**
WEN	*Diabelia serrata*	1	27.273	1.000	11	0	0	0.273	0.273	–	–	–	–	–
KAG1	*Diabelia serrata*	6	72.727	2.364	26	1	0	0.400	0.401	0.021	6	0.333	0.062	H8, H22
HYO	*Diabelia serrata*	1	36.364	1.273	14	0	0	0.364	0.364	–	1	–	–	H21
SHI	*Diabelia serrata*	9	72.727	2.727	30	0	0	0.290	0.417	0.287	8	0.000	0.000	H8
EHI1	*Diabelia serrata*	5	81.818	2.636	29	0	0	0.315	0.469	0.366	5	0.000	0.000	H1
SH1	*Diabelia serrata*	7	81.818	3.182	35	1	0	0.333	0.447	0.259	7	0.571	0.424	H1
SH3	*Diabelia serrata*	5	100	2.727	30	0	0	0.459	0.511	0.137	5	0.400	0.297	H1
SH7	*Diabelia serrata*	2	45.455	1.364	15	0	0	0.227	0.258	0.167	2	0.000	0.000	H1
SHA1	*Diabelia spathulata* var*. spathulata*	11	100	4.000	44	0	3	0.418	0.610	0.371	8	0.750	0.637	H10, H15
FUK2	*Diabelia serrata*	2	63.636	1.909	21	0	0	0.409	0.409	0	2	0.000	0.000	H9
TOK4	*Diabelia serrata*	3	72.727	2.545	28	0	0	0.394	0.539	0.316	3	0.667	0.124	H1, H6
GIF1	*Diabelia serrata*	1	54.545	1.545	17	0	0	0.545	0.545	–	1	–	–	H1
TOK5	*Diabelia serrata*	17	81.818	4.182	46	1	12	0.360	0.423	0.153	15	0.705	0.260	H2, H3, H5, H6, H11
TOK6	*Diabelia serrata*	20	90.909	5.091	56	2	10	0.437	0.571	0.244	19	0.456	0.339	H4, H6
OKA	*Diabelia serrata*	1	54.545	1.455	16	1	0	0.545	0.545	–	1	–	–	H8
KAG2	*Diabelia serrata*	25	90.909	4.273	47	0	10	0.377	0.475	0.211	13	0.500	0.136	H12, H13, H14
OIT	*Diabelia serrata*	13	81.818	3.091	34	0	5	0.360	0.422	0.151	1	–	–	H13
KAG3	*Diabelia serrata*	6	81.818	3.636	40	0	0	0.439	0.493	0.119	4	0.500	0.046	H16, H17
KOC3	*Diabelia serrata*	16	90.909	4.000	44	0	12	0.400	0.438	0.084	13	0.282	0.026	H19
KOC1	*Diabelia serrata* f. *buchwaldii*	19	90.909	6.091	67	5	20	0.499	0.614	0.192	16	0.000	0.000	H1
EHI3	*Diabelia serrata* f. *buchwaldii*	12	81.818	3.273	36	0	8	0.333	0.376	0.118	11	0.000	0.000	H20
NAG	*Diabelia serrata*	10	72.727	3.364	37	1	0	0.305	0.364	0.155	–	–	–	–
WEN2	*Diabelia serrata*	6	27.273	1.364	15	1	0	0.015	0.136	0.857	5	0.000	0.000	H16
YMS1	*Diabelia serrata*	–	–	–	–	–	–	–	–	–	1	–	–	H16
TOK7	*Diabelia serrata*	5	72.727	3.000	33	0	0	0.509	0.575	0.158	–	–	–	–
YAC	*Diabelia serrata*	7	90.909	2.818	31	1	0	0.429	0.417	−0.031	–	–	–	–
KOC4	*Diabelia serrata* f. *tomentosa*	1	45.455	1.455	16	0	0	0.455	0.455	–	–	–	–	–
YAM1	*Diabelia ionostachya* var*. ionostachya*	24	100	4.636	51	1	19	0.387	0.492	0.219	24	0.083	0.047	H28, H29
YAM2	*Diabelia ionostachya* var*. ionostachya*	15	90.909	5.000	55	12	11	0.176	0.546	0.683	15	0.000	0.000	H29
TOK3	*Diabelia ionostachya var. tetrasepala*	9	81.818	3.455	38	2	0	0.357	0.483	0.272	9	0.000	0.000	H13
YMS2	*Diabelia spathulata*	–	–	–	–	–	–	–	–	–	1	–	–	H24
SHI2	*Diabelia spathulata* var*. spathulata*	–	–	–	–	–	–	–	–	–	11	0.345	0.128	H18, H25, H31
SH4	*Diabelia ionostachya* var*. tetrasepala*	5	100	3.909	43	1	0	0.562	0.714	0.233	4	0.500	0.047	H25
MIE	*Diabelia ionostachya* var*. tetrasepala*	3	90.909	2.727	30	0	0	0.636	0.715	0.155	3	0.000	0.000	H25
SH2	*Diabelia spathulata* var*. spathulata*	8	100	4.000	44	0	0	0.537	0.617	0.129	8	0.250	0.023	H25
SH5	*Diabelia ionostachya* var*. tetrasepala*	3	81.818	2.636	29	0	0	0.455	0.485	0.077	3	0.667	0.062	H25
SH6	*Diabelia spathulata* var*. spathulata*	5	100	3.455	38	0	0	0.468	0.573	0.239	5	0.400	0.300	H25, H26
SHA2	*Diabelia spathulata*	2	72.727	1.909	21	0	0	0.455	0.485	0.111	2	0.000	0.000	H27
EHI2	*Diabelia spathulata* var*. spathulata*	2	90.909	2.273	25	1	0	0.636	0.576	−0.167	2	0.000	0.000	H7
SHI1	*Diabelia sanguinea*	11	90.909	3.727	41	0	5	0.376	0.518	0.281	6	0.533	0.050	H25, H30
KOC2	*Diabelia spathulata*	2	72.727	1.818	20	0	0	0.364	0.455	0.273	2	0.000	0.000	H18
KAN	*Diabelia spathulata* var*. spathulata*	1	45.455	1.455	16	0	0	0.455	0.455	–	1	–	–	H31
IBA	*Diabelia spathulata* var*. spathulata*	20	100	3.455	38	1	8	0.356	0.460	0.228	18	0.000	0.000	H31
FUK1	*Diabelia spathulata* var*. spathulata*	31	100	6.545	72	8	24	0.572	0.680	0.155	28	0.627	0.109	H18, H25
AIC	*Diabelia spathulata* var*. spathulata*	5	72.727	2.818	31	0	0	0.418	0.479	0.133	5	0.000	0.000	H33
NAR	*Diabelia spathulata* var*. spathulata*	1	45.455	1.455	16	0	0	0.455	0.455	–	1	–	–	H34
TOK1	*Diabelia spathulata* var*. spathulata*	2	81.818	2.091	23	1	0	0.500	0.576	0.217	3	0.667	0.062	H18,H35
TOK2	*Diabelia spathulata* var. *spathulata*	1	36.364	1.273	14	0	0	0.364	0.364	–	1	–	–	H18
GIF2	*Diabelia spathulata* var. *spathulata*	1	36.364	1.273	14	0	0	0.364	0.364	–	1	–	–	H25
WEN4	*Diabelia ionostachya* var*. wenzhouensis*	3	90.909	2.364	26	0	0	0.394	0.545	0.362	2	0.000	0.000	H23
WEN5	*Diabelia ionostachya* var*. wenzhouensis*	9	63.636	2.909	32	4	0	0.451	0.389	−0.167	7	0.286	0.111	H23, H37
NII	*Diabelia spathulata*	1	72.727	1.727	19	0	0	0.727	0.727	–	–	–	–	–
TOK8	*Diabelia serrata*	1	45.455	1.455	16	0	0	0.455	0.455	–	–	–	–	–
GYE	*Diabelia spathulata* var*. spathulata*	10	54.545	1.909	21	0	0	0.318	0.283	−0.133	7	0.000	0.000	H36
MIY2	*Diabelia sanguinea*	20	63.636	2.000	22	0	0	0.277	0.228	−0.225	13	0.000	0.000	H28
MIY1	*Diabelia sanguinea*	20	100	4.182	46	0	11	0.282	0.504	0.449	19	0.000	0.000	H28
TOC	*Diabelia sanguinea*	22	100	4.636	51	3	22	0.409	0.569	0.282	23	0.087	0.115	H28, H30
SAI	*Diabelia sanguinea*	25	90.909	3.636	40	0	11	0.331	0.461	0.287	24	0.000	0.000	H30
TOY	*Diabelia spathulata*	6	90.909	3.091	34	0	0	0.445	0.557	0.207	7	0.286	0.107	H29, H32

*P99, percentage of polymorphic loci when the most common allele had a frequency of <0.99; A, mean number of alleles per locus; TA, total number of alleles; PA, number of private alleles; RA, number of rare alleles (those occurring at frequencies below 0.05); Ho, observed heterozygosity; He, unbiased expected heterozygosity or Nei (1978) gene diversity. N, the number of individuals of the populations. Missing Fis, N2 cp—Hd cp and Hap definitions here*.

### DNA Extraction and Sequencing

Total genomic DNA was extracted from silica-dried leaf tissue using the modified cetyltrimethyl ammonium bromide (CTAB) protocol of Doyle and Doyle (1987). We amplified 11 nuclear microsatellite loci that were isolated from three species of *Diabelia* by Zhao et al. (2017). We selected two cpDNA intergenic spacer (IGS) regions (i.e., *rpl*32*-trn*L, *trn*H-*psb*A) which showed consistent amplification and have also been reported to be the most variable markers for subfamily Linnaeoideae (Wang et al., 2015). These markers allowed sampling of a larger number of individuals and testing of relationships between populations. The primers of these two chloroplast DNA regions are described in Dong et al. (2012) and Wang et al. (2015). Each polymerase chain reaction amplification for cpDNA and microsatellites was carried out in a 25 μL volume with the following reagents: Taq polymerase buffer, 10 ng total genomic DNA, 0.1–1 μl each of both forward and reverse primers, 12.5 μl 2 × Taq PCR MasterMix (Jinbaite, Biotechnology Co., Beijing, China), and ddH_2_O was added to make up the total volume of 25 μl. The thermal cycling conditions were 3 min at 95°C, followed by 43 cycles of 30 s at 95°C, 30 s at 54–58°C, and 1 min at 68°C, with a final extension of 20 min at 68°C.

The final PCR products (chloroplast DNA and microsatellite sequences) were purified with PEG8000 (Polyethylene Glycol) and sequenced using ABI Prism BigDye Terminator Cycle Sequencing Kits v. 3.1 on an ABI 3730xl DNA Sequencer (Life Technologies, 5791 Van Allen Way, Carlsbad, California 92008, USA) following the manufacturer's instructions.

### Genetic Analysis and Genotyping for Microsatellites

#### Alleles and Heterozygosity

GeneMarker 2.6.4 (LIZ500; Hulce et al., 2011) was used for Genotyping analysis. GenAlEx v. 6.1 (Peakall and Smouse, 2006) was used for estimating the standard genetic diversity parameters at both population and species levels for: percentage of polymorphic loci when the most common allele had a frequency of <0.99 (%P_99_); mean number of alleles per locus (*A*); observed heterozygosity (*H*_o_); unbiased expected heterozygosity or Nei (1978) gene diversity (H_*e*_); genetic diversity indices of the populations of *Diabelia*; sum of squared variation within populations; and source of variation. GenePop v.4.2.2 (Raymond and Rousset, 1995b) was used for checking genotypic linkage disequilibrium between pairs of loci at the population level and across all populations, but also for calculating possible deviations from Hardy-Weinberg (H–W) equilibrium within each population and for each locus per population. For both calculations, GenePop used a Fisher's exact test following the Markov chain (MC) algorithm (Raymond and Rousset, 1995a). The frequency of null alleles was estimated by following the expectation maximization (EM) algorithm (Dempster et al., 1977) in Free NA (Chapuis and Estoup, 2006). Free NA was also used to estimate *F*_ST_ values between pairs of populations and species (with and without the method of excluding (ENA) correction for null alleles; Chapuis and Estoup, 2006). Pairwise genetic distance between populations was calculated using two algorithms, Nei (1972) standard genetic distance (D_S_) and Roger's (1972) distance (D_R_). These distance matrices were converted into UPGMA (unweighted pair-group method using arithmetic averages) dendrograms (after 1,000 bootstrap replicates) employing the programs Populations v.1.2.30 (Langella, 1999) and TreeView v.1.6 (Page, 1996).

### Population Genetic Structure for Microsatellite Sequence Data

The genetic structure was assessed through five different methods:

First, STRUCTURE v. 2.3.4 (Pritchard et al., 2000), a widely-employed clustering software that is based on a Bayesian algorithm, was used. On the basis of preliminary runs, *K* was run from 1 to 20 (10 independent runs were run for each *K* value) assuming an admixture model with correlated allele frequencies, and with a priori grouping of individuals into populations (but not into species). The length of the burn-in period and the MCMC runs were set to 50,000 and 500,000, respectively. The most likely value of *K* was determined both by choosing the smallest K after the log probability of data [ln Pr(X|K)] values reached a plateau (Pritchard et al., 2000) and by the ΔK statistic of (Evanno et al., 2005) with the aid of Structure Harvester v. 0.6.94 (Earl, 2012). For the most likely K, Clumpp v. 1.1.2b (Jakobsson and Rosenberg, 2007) was used to combine the results of the 10 independent runs of the best K. To plot the output result produced by Clumpp, we used the program Distruct v. 1.1.

Second, a molecular variance analysis (AMOVA) was run with the aid of GenAlex and Arlequin, calculating the fixation indices and percentage of molecular variance at four hierarchical levels and the percentage of molecular variance at five hierarchical levels (based on the structure results of nSSR; R1, R2, R3, R4, R5): (i) among species; (ii) among populations; (iii) calculation of the sum of squared variation within populations; and (iv) intraspecific variance. Similar to the first level, several possible combinations of populations were used, and based on the STRUCTURE analysis the clusters were divided into five geographic regions: (A), West Japan; (B), South Japan (including Nara and Wakayama); (C), Southeast Japan (including Ibaraki, Chiba, Kanagawa); (D), Northwest Japan (including Akita, Yamagata, Niigata), China (Wenzhou) and Korea (Gyeongsangnam-do); and (E), Northeast Japan (including Fukui, Shiga, Kyoto); where the study populations occurred (Japan, Korea, and China), and the four species (*D. serrata, D. spathulata, D. ionostachya*, and *D. sanguinea*). Third, a Principal Coordinates Analysis (PCoA) at the population level was carried out with GenAlEx. Fourth, putative genetic barriers between populations were detected with the software Barrier v. 2.2 (Manni et al., 2004); significance of identified barriers was tested by bootstrapping 1,000 Nei's genetic distance *D*_A_ (Nei and Takezaki, 1983) matrices that were previously obtained with Microsatellite Analyzer (MSA) v. 4.05 software (Dieringer and Schlötterer, 2003). Fifth, a Mantel test (1,000 permutations) between the pairwise population matrix of Nei Genetic Identity and the matrix of the log-transformed geographical distances was carried out using the software GenAlEx.

Gene flow was estimated with two time-frameworks. First, we estimated recent (i.e., within the recent 2–3 generations) migration rates between the clusters identified with STRUCTURE; between the three geographical regions where the populations under this study occurred; and between the four species using a Bayesian assignment test with the software BayesAss v. 3.0 (Wilson and Rannala, 2003). A total of 2 × 10^7^ MCMC iterations were run, with a burn-in of 2 × 10^6^ iterations and a sampling frequency of 2,000; while the mixing parameters deltaA, deltaF, and deltaM were set to 0.40, 0.60, and 0.20. The convergence and stability of the MCMC algorithm were checked by visual inspection of plotted posterior parameter estimates using the software Tracer v. 1.6 (Rambaut et al., 2014). Second, historical mutation-scaled migration rates (*M* = *m/*μ, where *m* is migration rate and μ is mutation rate per generation) were estimated by using MIGRATE-N v. 3.6.4 (Beerli and Felsenstein, 2001) with the same sampling scheme as that of BayesAss. Ten replicates were run under a Brownian motion model, assuming a constant mutation rate for all the loci. With a Bayesian approach, a long chain with 20,000 genealogies to sample was run, with a sampling increment of 100 (thereby generating 2,000,000 genealogies for each replicate); the burn-in was set to 20,000. A static heating scheme was chosen (temperatures were specified to 1.00, 1.50, 3, and 1 × 10^6^), with uniform prior distribution both for Θ and *M* (min: 0; max: 100; delta: 10). The effective number of migrants per generation (Nm) among populations was estimated using the formula 4 *Nm* = ΘM (Beerli and Felsenstein, 2001). Total immigration and emigration rates for each population were obtained by summing values of *Nm*. The analyses were carried out at the CIPRES bioinformatic facility (Miller et al., 2010).

### Phylogenetic Analyses Based on cpDNA

DnaSP v. 5 (Librado and Rozas, 2009) was used to determine haplotypes (based on two chloroplast fragments *rpl*32*-trn*L and *trn*H-*psb*A from 402 individuals; Figure S1).

**Figure 2:**
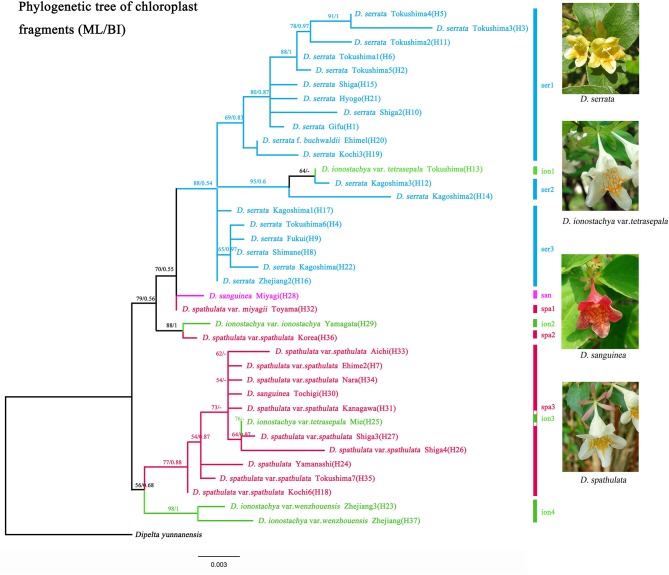
Maximum Likelihood tree inferred from RAxML for 37 haplotypes in *Diabelia* based on concatenated sequences of chloroplast DNA (*trn*H*-psb*A*, rpl*32*-trn*L) with *Dipelta yunnanensis* as the outgroup. Numbers associated with branches are ML bootstrap support values. Population migration direction inferred (a, b, and c) from haplotype phylogenetic tree of *Diabelia*.

We edited the sequences and assembled them with Sequencher v. 4.7 (Gene Codes Corporation, Ann Arbor, Michigan, USA). We aligned the newly generated sequences and sequences from GenBank with Clustal W implemented in MEGA X version 10.0.5 software (Kumar et al., 2018) and they are further manually adjusted with Se-Al v. 2.0 (Rambaut, 1996). Prior to concatenating the dataset of each marker, incongruence length difference (ILD) tests were performed on two datasets. We concatenated the datasets with SequenceMatrix (Vaidya et al., 2011). All the sequences identified in the present study were deposited in GenBank (Table S1). Phylogenetic analyses were independently performed for the two chloroplast markers (*psb*A*-trn*H and *rpl*32*-trn*L), representative sample sequences of haplotypes are shown in Figure 2. As *Dipelta* is a sister genus of *Diabelia* (Wang et al., 2015), we took *Dipelta* as an outgroup for the phylogenetic tree construction in this study.

The evolutionary model (best of fit) that was most appropriate for all the data was selected according to the corrected Akaike Information Criterion (AICc) implemented in jModelTest (Santorum et al., 2014). The probabilistic analysis was conducted using RAxML (v. 8) (Stamatakis, 2014) for maximum likelihood (ML) using the default parameters with bootstrap support of 1,000 pseudo-replicates and MrBayes v. 3 (Huelsenbeck and Ronquist, 2001) for Bayesian inference with 5 × 10^5^ generations with two runs and four chains following the substitution matrix assessed as mentioned above. Both analyses were performed on the CIPRES Science Gateway website (Miller et al., 2010) and cladograms were edited with the program TreeGraph2 beta v. 2.0 (Stöver and Kai, 2010).

### Ecological Niche Modeling of *Diabelia*

Ecological niche modeling (ENM) was performed to evaluate the potential distribution of the four extant species of *Diabelia* under present climatic conditions and projected back to the Last Glacial Maximum (LGM, ca. 21,000 year BP). We employed the maximum entropy algorithm, as implemented in MaxEnt v. 3.3 (Phillips et al., 2006). The current distribution information for the four *Diabelia* species was obtained from Hara (1983) and Landrein and Farjon (2019). Data were based on specimens deposited in the main Chinese herbaria (through the Chinese Virtual Herbarium platform; www.cvh.ac.cn), from the collection records of Laboratory for Plant Systematics in Chiba University (bean.bio.chiba-u.jp/eng/index.php; Table S2). In total, after removing duplicate records within each pixel (2.5 arc-min, ca. 5 km), we obtained 67, 67, 69, and 31 records for *D. serrata, D. spathulata, D. ionostachya*, and *D. sanguinea*, respectively. A set of 19 bioclimatic variables at 2.5 arc-min resolution covering the distribution range (and neighboring areas) for all four species under current conditions (1950–2000) were downloaded from the WorldClim website (www.worldclim.org; Hijmans et al., 2005). After a correlation analysis of the bioclimatic variables within the study area (with the help of SDM toolbox v. 1.1b; Brown, 2014, we selected a smaller set of 13 (relatively) uncorrelated variables: mean diurnal range (bio2), isothermality (bio3), temperature annual range (BIO5-BIO6) (bio7), mean temperature of the wettest quarter (bio8), mean temperature of the driest quarter (bio9), mean temperature of the warmest quarter (bio10), mean temperature of coldest quarter(bio11), annual precipitation (bio12), precipitation of wettest month (bio13), precipitation of the driest month (bio14), precipitation seasonality (bio15), and precipitation of the warmest quarter (bio18), precipitation of coldest quarter (bio19). The selection of variables from pairs or groups of highly correlated (*r* ≥ 0.9) variables were made based on their relative contribution to the model (percent contribution, permutation importance jackknife of regularized gaining train), making sure that the most influential variables for the two outgroup species were selected.

The distribution model under current conditions was projected to the LGM using palaeoclimatic layers simulated by the Community Climate System Model version 4 (CCSM4; Gent et al., 2011), the Model for Interdisciplinary Research on Climate Earth System Model (MIROC-ESM; Watanabe et al., 2011), and the New Earth System Model of Max Planck Institute for Meteorology (MPI-ESM; http://www.mpimet.mpg.de/en/science/models/mpi-esm/). Replicate runs (20) of MaxEnt (using the “subsample” method) were performed to ensure reliable results. Model performance was assessed using the area under the curve (AUC) of the receiver operating characteristic plot, with 25% of the localities randomly selected to test the model. AUC scores may range between 0.5 (randomness) and 1 (exact match), with those above 0.9 indicating good performance of the model (Swets, 1988). The MaxEnt jackknife analysis was used to evaluate the relative importance of the 13 bioclimatic variables employed, based on their gain values when used in isolation. To convert the continuous value projection to a binary presence/absence distribution, we applied the maximum sensitivity plus specificity logistic threshold, which is very robust with all types of data (Liu et al., 2016). All ENM predictions were visualized in ArcGIS v.10.2 (ESRI, Redlands, CA, USA).

## Results

### CpDNA Diversity and Phylogenetic Results of *Diabelia*

The total alignment of the two chloroplast regions (*trn*H*-psb*A*, rpl*32*-trn*L) surveyed across the 402 individuals of *Diabelia* was 1,127 bp long. Thirty-seven haplotypes (H1–37) were identified across the 51 populations of *Diabelia* (Figure 1). Four haplotypes (H13, H25, H28, and H29) were found to be shared by multiple species. H25 was shared haplotype of *D. ionostachya, D. sanguinea*, and *D. spathulata*. H13 was a shared haplotype between *D. ionostachya* and *D. serrata*. H28 was a shared haplotype between *D. ionostachya* and *D. sanguinea*, while H29 was the shared haplotype between *D. ionostachya* and *D. spathulata* (Figure 1).

As currently defined, *Diabelia serrata* has 18 haplotypes, mainly distributed in the southwest of Japan, with diversity ranging from 0.282 to 0.75 (cpDNA, Table 2). We found 15 haplotypes in *D. spathulata*, mainly distributed in central and southern Japan, and the diversity ranged from 0.25 to 0.667 (cpDNA, Table 2). There were three haplotypes (H25, H28, and H30, Figure 1) in *D. sanguinea*, mainly distributed in the northeast of Japan (e.g., Akita, Toyama), and the diversity ranged from 0 to 0.087 (cpDNA, Table 2). There were six haplotypes (H13, H23, H25, H28, H29, and H37) in *D. ionostachya*, distributed in Wenzhou (China) and Saitama (Honshu), and the diversity ranged from 0 to 0.083 (Figure 1; Table 2).

Based on the two cpDNA regions (*rpl*32*-trn*L, *trnH-psbA*), *D. serrata* formed a monophyletic group (BS = 88, PP = 0.54, Figure 2; Figure S1) with the inclusion of one sample of *D. ionostachya* var. *tetrasepala* (H13). The first diverging clade included *D. serrata* in Kagoshima (Table 1, KAG3; haplotype H17), and Zhejiang (Table 1, WEN2; haplotype H16, Figure 1). *Diabelia spathulata, D. ionostachya*, and *D. sanguinea* were not monophyletic in these analyses, with intermixed samples forming a series of clades that were sequentially sister to *D. serrata*, though the exact relationships between some clades are only weakly supported (Figure 2; Figure S1).

*Diabelia ionostachya* var. *wenzhouensis* Landrein from China was also weakly supported as sister to *D. spathulata* in Japan (Figure 2; Figure S1, Bootstrap value (BS) = 56, PP = 0.68). *Diabelia spathulata* in Nara, Ehime, Aichi and Kochi Prefectures (H7, H18, H33, and H34) were found to be sister to *D. ionostachya* var. *wenzhouensis* (H37), sharing a common ancestor. *Diabelia spathulata* showed a pattern of ancient divergence between populations. The earliest diverging clade of *D. spathulata* in Japan is distributed in Kochi (Figure 2, *D. spathulata* var. *spathulata* Kochi6 (H18). *Diabelia spathulata* from Korea formed a clade with *D. ionostachya* var. *ionostachya* from Yamagata (BS = 88, PP = 1) (Figure 2), likely representing a dispersal event between Japan and Korea.

### Genetic Diversity Based on NSSR

A total of 549 individuals from 66 populations across the range of the genus were genotyped with nSSR (some representative individuals were selected). All the 11 surveyed microsatellites were polymorphic across populations. Significant linkage disequilibrium was detected in 158 out of 1,207 locus–locus comparisons (about 13%), although only nine persisted after the implementation of Bonferroni's correction (Rice, 1989). A significant deviation from H–W equilibrium expectations (*P* < 0.05) was observed for 117 of 549 validity tests, mostly showing a higher than expected frequency of homozygosity. The values of null allele frequency at all loci were very low, averaging 0.051 [range = 0.027 (locus 8)−0.114 (locus 7)]; low percentages of null alleles are not expected to cause significant biases in genetic diversity estimates (*cf*. Dakin and Avise, 2004; Orsini et al., 2008). The differences between the “raw” values of *F*_ST_ (one of the most sensitive parameters when null alleles occur; Chapuis and Estoup, 2006; Chapuis et al., 2008) and those after correcting for the presence of null alleles in our dataset were negligible (<3%). Nevertheless, there was a positive correlation between the frequency of null alleles and *F*_IS_ values per locus within (*N* = 356, *R*^2^ = 0.709, *P* = 0.000) and across populations (*N* = 11, *R*^2^ = 0.701, *P* = 0.001), indicating that the significant positive deviations from H–W equilibrium can be at least partly attributed to the presence of null alleles.

A total of 187 alleles were detected within the study system, ranging from 10 (locus 9) to 25 (locus 11) and averaging 17 alleles per locus (Table 2). The number of polymorphisms was only slightly higher for populations of *D. sanguinea* and *D. ionostachya* compared to *D. serrata and D. spathulata*, both for allelic richness and for heterozygosity (Table 3), even though sample numbers for *D. sanguinea* and *D. ionostachya* were lower. Indeed, polymorphism values only increased moderately for parameter *A* (number of alleles) when only populations with *N* > 10 were considered (Table 2). The most variable populations belonged to *D. ionostachya* and are distributed in the Northeast region and Tokushima. However, the richest populations in terms of alleles were *D. spathulata* from Fukui (SP-FUK1) (highest values of *A* and *TA*; Total number of alleles) and *D. spathulata* from Yamagata (SP-YAM2) (the population with most private alleles), whereas the population with the largest value of *H*_e_ was also SP-FUK1. Populations outside Japan showed low genetic diversity, especially *D. serrata* (the only non-Japanese population) (WEN) (*H*_e_ = 0.273) (Table 2).

**Table 3:** Analysis of molecular variance (AMOVA) and gene-frequency of *Diabelia* within species, areas and total based on nSSR and chloroplast DNA variation.

**Population**	***P* (99%)**	***A***	***TA***	***PA***	***Ho***	***He***	***Fis***
*Diabelia serrata*							
Mean (all populations)	72.727	2.899	31.89	0.48	0.386	0.377	−0.107
Mean (only populations *N* > 10)	86.869	4.152	45.667	0.55	0.388	0.46	0.148
Species level	100	12.091	133	33	0.387	0.641	0.395^***^
*D. spathulata*							
Mean (all populations)	69.008	2.45	26.95	0.727	0.435	0.354	−0.388
Mean (only populations N > 10)	87.273	3.764	41.4	2.2	0.376	0.453	0.149
Species level	100	10.636	117	18	0.431	0.726	0.416^***^
*sanguinea*							
Mean (all populations)	89.394	3.545	39	0.333	0.353	0.454	0.199
Mean (only populations *N* > 10)	89.091	3.636	40	0.399	0.335	0.443	0.208
Species level	100	7.909	87	2	0.34	0.644	0.474^***^
*D. ionostachya*							
Mean (all populations)	81.818	3.025	33.27	1.727	0.405	0.427	−0.004
Mean (only populations *N* > 10)	90.909	4.212	46.33	4.664	0.29	0.45	0.39
Species level	100	10.909	120	19	0.361	0.739	0.529^***^
Mean genus *Diabelia*	2.829				0.403	0.385	

*A, mean number of alleles per locus; TA, total number of alleles; PA, number of private alleles; Ho, observed heterozygosity; He, unbiased expected heterozygosity or Nei (1978) gene diversity; F_IS_, inbreeding index*.

*** *p <0.001*.

**Figure 3:**
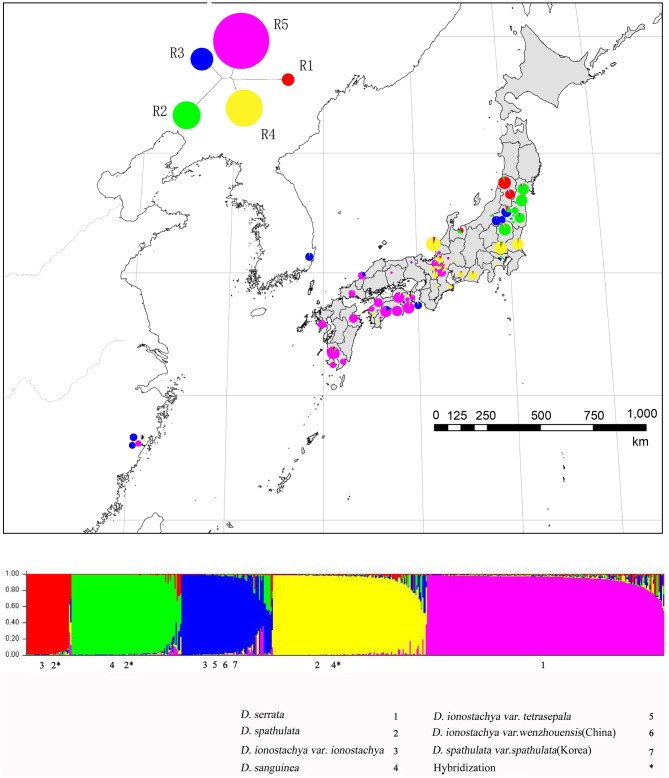
Results of STRUCTURE analysis for all individuals of *Diabelia* studied based on nSSR data. Assignment of individuals to genetic clusters at *K* = 5.

The genetic differentiation within populations of *Diabelia* based on *F*_ST_ was significant [mean *F*_ST_ (66 populations) = 0.419; Table 4]. The *F*_ST_ values for individual species were also higher (mean *F*_ST_; *D. serrata* = 0.288; *D. spathulata* = 0.358; *D. sanguinea* = 0.388, *D. ionostachya* = 0.558. The difference between the lowest and the highest *F*_ST_ was 0.27 (*D. serrata, F*_ST_ = 0.288; *D. ionostachya, F*_ST_ = 0.558) showing low interspecific differentiation. The “among-species” component was also low (8.9%) in AMOVA (Table 4). According to STRUCTURE results of the nSSR data, the mean *F*_ST_ (66 populations) was found to be 0.437 based on AMOVA analysis. The *Fst* value of each gene-pool was lower than the four species concept (R1 = 0.075; R2 = 0.217; R3 = 0.474; R4 = 0.234; R5 = 0.217, Figure 3). The difference between the lowest and highest *F*_ST_ value was 0.399. The gene pool differentiation was also very high (22.7%; Table 4). The Mantel test showed a low *P*-value (0.010), indicating a significant correlation between genetic differentiation and geographical distance, thereby suggesting that there is an isolation-by-distance across all studied populations. The greatest variation was found among all the populations of *D. serrata* (1568), followed by the populations of *D. spathulata* (1082), *D. sanguinea* (762), and *D. ionostachya* (710), respectively. The population of *D. spathulata* from Fukui (SP-FUK1) had the highest mutation value (240), followed by the population of *D. sanguinea* from Tochigi (SA-TOC; 137) and the population of *D. serrata* from Kagoshima (SR-KAG2; 136). At the population level, the four species were found to be diverging at different rates, and *D. ionostachya* was more variable than other species (Tables 3, 4).

**Table 4:** *F*-statistics of the five gene-pool (the result of STRUCTURE-nSSR) and four species within *Diabelia*.

**Population**	**Fst**	**Fsc**	**Fct**	**Percentages of molecular variance**		
**Lineage**				**Among species(subspecies/varieties)**	**Among populations within species**	**Within populations**
R1	0.075	–	–	–	7.50%	92.50%
R2	0.217	–	–	–	21.70%	78.30%
R3	0.474	0.43	0.078	7.80%	39.70%	52.60%
R4	0.234	0.152	0.096	9.60%	13.70%	76.60%
R5	0.217	0.158	0.07	7.00%	14.70%	78.30%
*Diabelia* (*K* = 5)	0.437	0.271	0.227	22.70%	21.00%	56.30%
*Taxon*						
*D. sanguinea*	0.388	–	–	–	38.80%	61.20%
*D. serrata*	0.288	–	–	–	28.80%	71.20%
*D. spathulata*	0.358	–	–	–	35.80%	64.20%
*D. ionostachya*	0.558	–	–	–	55.80%	44.20%
*Diabelia (K = 4)*	0.419	0.362	0.089	8.90%	33.00%	58.10%

*Fst, the fixation index within populations relative to the total; Fsc, genetic differences among species (possible subspecies/varieties) defined a priori; Fct, genetic differences among population within species (possible subspecies/varieties)*.

The results of STRUCTURE analyses for all individuals based on the nSSR markers are presented in Figure 3; Figure S2. According to Evanno's approach and plot of mean posterior probability values of each *K* (Figure S3), *K* = 5 was the most likely number of genetic clusters for 0.5 million generations. The genetic clustering was highly different from *K* = 1 to *K* = 25. However, *K* = 9 was the most likely number of genetic clusters for 1 million generations. The genetic clustering was highly different from *K* = 1 to *K* = 20. Comparing the two graphs of *K* = 5 and 9 (Figure S2), the cluster diagram of the 1 million generation of *K* = 9 is more complex than that of the 500,000 generations, perhaps the results of *K* = 9, in theory, will be more refined, but more refined results also require greater sampling to support these analyses and it would be more persuasive to have at least 1,000 samples of the data in the 1 million generations of *K* = 9, as our analyzed sample set is too small to give accurate results. Secondly, with more iterations, uncertainty factors may be also magnified and produce inaccurate results. In addition, the results of the 1 million generations of *K* = 9 does not reflect morphological trait diversity. In contrast, the results of the 500,000 generations *K* = 5 take into account the size of the cluster and the gene pool in the PCoA (Figure S2), identifying four large clusters and a small red cluster representing an unusual gene pool in the northernmost part of Japan. This small cluster may represent a relatively primitive area of diversity; however, this is difficult to assess as it is represented by relatively few samples. In summary, it is better to merge the possible additional lineages represented by *K* = 9 into five larger lineages, consistent with the results of our phylogenetic analyses (Figure S2).

The distribution of all *Diabelia* populations in five distinctive lineages are presented in Figure 3. *Diabelia serrata* is a distinct lineage (R5; Figure 2). However, *Diabelia spathulata* (R4) as well as *D. ionostachya* var. *ionostachya* (R1; Figure 2). *D. ionostachya* var. *wenzhouensis, D. ionostachya* var. *tetrasepala* and *D. spathulata* var. *spathulata* from Korea form another distinct lineage (R3; Figure 2). The result of this structure (*K* = 5) does not match current taxonomic concepts (Hara, 1983; Landrein, 2010). Samples of *D. sanguinea* (R2; Figure 2) were often admixed with *D. spathulata* or *D. ionostachya*. R1 is mainly located in the Yamagata area of northwestern Japan, Southward in Fukushima, Toyama, Tukui, and nearby regions, and hence there is a degree of introgression. R2 is mainly located in Miyagi, Fukushima, Tochigi area of northeastern Japan, south in Saitama, Tokyo, Shizuoka, and Shiga Prefectures and shows minor introgression. R3 is relatively scattered, occurring in Chinese and Korean populations, but mainly in the northeastern part of Japan in Fukushima Prefecture. Populations in Japan's Tokushima, Kochi, Shimane Prefectures showed low introgression. R4 is mainly located in the central part of Honshu Prefecture, Japan, to the eastern Pacific coast and to Ibaraki Prefecture with scattered populations in the north of Tochigi, Fukushima, Miyagi, and nearby Prefectures show minor introgression. R5 is primarily found in Shikoku and Kyushu Prefectures in southern Japan, and the central region of Honshu, with a limited occurrence in Zhejiang, China. It should be noted that due to the limited number of samples in some clades, the conclusion of *K* = 5 taxonomic entities requires further investigation with more detailed sampling.

### Ecological Niche Modeling Based on *Diabelia* Location Information

**Figure 4:**
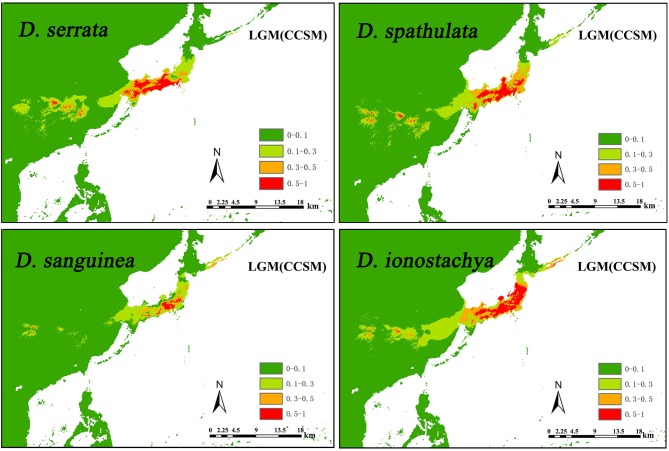
Comparison of potential distributions as probability of occurrence for *Diabelia serrata, D. spathulata, D. sanguinea*, and *D. ionostachya* (Landrein and Farjon, 2019), at the CCSM climatic scenarios of the Last Glacial Maximum (LGM, ca. 21,000 years BP). The maximum training sensitivity plus specificity logistic threshold has been used to discriminate between suitable (cutline 0.1–1 area) and unsuitable habitat. The darker color indicates a higher probability of occurrence.

The AUC scores averaged across 10 runs were higher for the four species (mean ± SD, 0.997 ± 0.001 for *D. serrata*, 0.997 ± 0.002 for *D. spathulata*, 0.998 ± 0.001 for *D. sanguinea*, and 0.996 ± 0.001 for *D. ionostachya*), which supported the predictive power of the model. According to the MaxEnt jackknife tests of variable importance, the precipitation variables were more informative for the model than the temperature ones under the present climate conditions (bio 14, bio 15, bio 18, and bio 19 were invariably very informative for the four species), although bio9 (mean temperature of driest quarter) was also important for all the models. The present-day distributional predictions for the four species were largely congruent with the known occurrences, although additional areas appeared as suitable (cutline 0.1–1 areas in Figure 4; Figure S4), including some areas in eastern and south-eastern China, and for *D. spathulata*, a thin coastal strip in South Korea. Projections of the species niche to the LGM climate produced considerably different maps of the probability of occurrence at the LGM compared to the present time. In general, with the two LGM models, large areas appeared as suitable in mainland China for all species, although the suitability of the exposed East China Sea shelf and Japan greatly varied depending on the model and the species (Figure 4; Figure S4). For four species of *Diabelia*, the distribution of the two LGM models is very similar; species had a potentially continuous range through most of south-eastern/eastern mainland China, and the contiguous exposed East China Sea shelf (Figure 4; Figure S4). For the CCSM and MIROC model (MIROC model simulates somewhat warmer and dryer LGM conditions compared to CCSM within the study area; mean annual temperature, −22.2 to 26.4 °C (average, 3.6°C) for CCSM and −11.0 to 25.4°C (average, 8.5°C) for MIROC; annual precipitation, 70–5,531 mm/year (average, 921 mm/year) for CCSM and 58–3,982 mm/year (average, 549 mm/year for MIROC). The distribution density and range of *Diabelia* under the MIROC model were slightly lower than the distribution density and range under the CCSM model, but the differences were not significant. The potential range for *Diabelia* species in mainland China would have been much smaller, while habitats would have remained stable across most of the current distribution area within Japan (Figure 4; Figure S4).

## Discussion

### CpDNA Genetic Diversity and Regional Population Genetic Structure of *Diabelia*

In terms of genetic diversity, the geographic distribution of *Diabelia* species is highly discontinuous (Figure 1). However, populations of *Diabelia* species still show similar or even higher diversity at haplotype (cpDNA) and nucleotide levels (*Ht* = 0.926; πt = 0.0072; Tables 2, 3) than that of other endemic populations of China and Japan [e.g., *Croomia japonica* and *C. heterosepala*, (Li et al., 2008); *Dysosma versipellis*, (Qiu et al., 2009a)]. *Diabelia* species have high microsatellite genetic diversity (*He* = 0.736; Tables 2, 3). High genetic diversity usually means a very long evolutionary history (Huang et al., 2001; Qiu et al., 2009b). Wang et al. (2015) estimated chronogram of Linnaeoideae and its relatives based on nine plastid markers data, infers that the species differentiation in *Diabelia* occurred in the Miocene (5–23 Ma), when the genus may have been widely distributed in southern Asia. However, after the Quaternary Period climate shocks and more recent environmental constraints, the main distribution of *Diabelia* in Asia was confined to Japan. The main distribution in southern China (during the LGM period) had disappeared except in Zhejiang, and a large number of populations are likely to have gone extinct (Qiu et al., 2011; Qi et al., 2012; Sakaguchi et al., 2012; Zhai et al., 2012). In the phylogenetic tree based on chloroplast markers, *Diabelia* shows a typical multi-origin distribution pattern (Figure 2; Figure S5). Therefore, the high genetic variability of *Diabelia* may also be due to the inheritance of variation from ancestral populations. Based on the genetic diversity analysis of microsatellite markers, we found relatively low genetic variation at the population level (mean *He* = 0.385, Table 3). Meanwhile, the microsatellite analysis of *Diabelia* revealed a high degree of genetic differentiation (*Fst* = 0.419; Table 4). Similar to *Diabelia, Cercidiphyllum*, a Chinese-Japanese discontinuous species, has similar genetic characteristics (Qiu et al., 2011; Qi et al., 2012; Sakaguchi et al., 2012; Zhai et al., 2012).

The above population genetic characteristics may reflect certain biological characteristics of *Diabelia*. In Japan, the main distribution area, *Diabelia* population-level habitat fragmentation due to climatic variation may have driven current disjunctions (Fu and Jin, 1992). Through bottleneck analysis, we found that only four of the 36 Japanese populations with more than five individuals had experienced recent bottlenecks under the IAM model, indicating that Japanese populations had not experienced serious bottleneck effects during recent climatic variations, which was similar to the previous distribution pattern (ENM, Figure 4). In terms of breeding system, although *Diabelia* has relatively small winged seeds that can be dispersed by wind (Hufford, 2004), its small size, growth at particular altitudes and undergrowth habitats may significantly reduce the likelihood of dispersal over long distances. Based on the above hypothesis, although *Diabelia* is widely distributed in Japan and has not experienced serious bottleneck effects in recent history, it shows low genetic diversity and high genetic differentiation among populations. The population genetic history of *Diabelia* suggests that interruption of gene flow and genetic drift are the main factors driving existing population structures in Japan.

### Potential Refugia and Demographic History

The glacial and interglacial fluctuations caused by Quaternary climate shocks have a great influence on the distribution of species (Comes and Kadereit, 1998; Hewitt, 2000). Most plant distributions undergo expansion and contraction, leading to population migration or extinction, followed by geographical isolation, differentiation and subsequent population expansion (e.g., Liu et al., 2013). Wang et al. (2015) estimated that species differentiation in *Diabelia* occurred in the Miocene (5–23 Ma), suggesting that extant populations likely differentiated well before the LGM. Compared with the LGM, the Tertiary climate and the relative location simulation of the ECS, China and Japan are not developed enough to predict the role of the ECS in the species differentiation of *Diabelia*. Although the phylogenetic tree shows that the populations of China and Japanese have significant genetic differentiation (Figure 2). The climate shocks in the LGM period may have had a significant impact on the recent history of *Diabelia* at a population level compared with the previous period of species differentiation (Qiu et al., 2011; Qi et al., 2012; Sakaguchi et al., 2012, 2017; Zhai et al., 2012). In the haplotype network, the primitive haplotypes associated with *Diabelia* are determined by inserting an outgroup (Figure 1, *Dipelta yunnanensis* BOP012201). In the haplotype network in *Diabelia*, haplotype H23, H29, H36, H37 were found to be connected with haploidy of external groups (Figure 1). It was found that the distribution of ancestral haploidy was very scattered in geographical location, in Zhejiang Province of China, Gyeongsangnam-do of Korea, and Yamagata County of Japan, respectively, suggesting that *Diabelia* had an extensive distribution in southeast China and Japan (excluding Hokkaido) during the LGM. The predicted results of ENM in LGM also support this conclusion. The distribution records of *Diabelia* in China are all from one area in Zhejiang Province. In addition, plants growing in glacial refugial areas are generally expected to exhibit corresponding ecological characteristics, such as low dispersal capacity, niche specialization (Hopper, 2009) and harbor unique haplotypes and a relatively higher level of genetic diversity than populations not influenced by glaciation (Comes and Kadereit, 1998; Petit et al., 2003). The populations in Zhejiang Province, which have not been dispersed to other areas, have unique haplotype (H23, H37, Figure 1) and high genetic diversity (WEN5, cp-π: 0.111; Table 2). Another group (WEN4) is represented by only two individuals which do not allow for meaningful assessment. During the Quaternary period, although China's subtropical region experienced severe climatic shocks during the glacial and interglacial periods, large areas of the region remained unfrozen during the LGM period (Axelrod et al., 1996) with many refuge zones for ancient and relict species (e.g., Wang et al., 2009). After the LGM, *Diabelia* populations in southeastern China may have experienced a large-scale population extinction event, and Zhejiang may be a glacial refuge for *Diabelia ionostachya* var. *wenzhouensis*. Our data support the Chinese population as more divergent than other lineages, and it is not the result of a recent dispersal from Japan. At the same time, our ENM results also provide strong evidence for survival of *Diabelia* in southern China during the LGM period (Figure 4; Figure S4). Similar to the population in China, the population in South Korea also had primitive haplotypes (H36; Figure 1), but did not exhibit high genetic diversity and high genetic differentiation as would be expected for a refugial population (Hewitt, 2000). The degree of genetic diversity was very low (GYE, 0; Table 1), and Bottleneck analysis shows that Korean *Diabelia* population has experienced a very serious bottleneck effect in its recent history (Table S3). The genetic relationship between the two populations, though far apart from each other, was very close, and both are derived from a common ancestor in our phylogenetic analysis (Figure 2). During the LGM period, it appears there was no population of *Diabelia* in the Korean Peninsula based on ENM (Figure 4). The population in Korea is more likely the result of very recent long-distance transmission across the Tsushima Strait from northern Japan. Korean populations are related to *D. ionostachya* not *D. spathulata* which is accordance with the previous conclusion by Shin et al. (2012).

The altitudinal ranges of species in *Diabelia* are relatively narrow, but could potentially have expanded significantly during the LGM. *Diabelia sanguinea* is a higher altitude species (Landrein and Farjon, 2019). In China and East Asia, although most areas were not covered by ice sheets, violent fluctuations in the Quaternary Ice Age also affected the distribution and evolutionary history of flora and fauna in the region (Axelrod et al., 1996). The genealogy of five different alpine plants in Japan was studied by Fujii and Senni (2006), and it was inferred that alpine plants had shelters for warm interglacial periods in the alpine areas in the middle of Honshu Island. Several studies of the endemic Fagus crenata (altitude 600–1,900 m) in Japan have shown that its current distribution pattern formed after the Ice Age by the migration of multiple shelters from the Sea of Japan and the Pacific Rim (Tomaru et al., 1998; Fujii et al., 2002; Okaura and Harada, 2002). Most pine fir sanctuaries are located along the southwest Pacific coast, spreading northward after the Ice Age (Ikeda et al., 2006; Tsumura, 2006). The study on the phylogeography of *Potentilla matsumurae* (altitude 700–3,100 m) further confirmed the inference that the alpine mountains in the middle of Honshu Island were refugees of the interglacial age (Fujii and Senni, 2006). Fujii and Senni (2006) found that Japan's alpine plants have experienced a different evolutionary history from European alpine plants during the interglacial periods because of the expansion of forests, so they retreated to refugia, both in the mountains, and at low altitudes (Fujii et al., 1997, 1999; Fujii and Senni, 2006; Ikeda et al., 2006; Sakaguchi et al., 2012, 2017; Zhai et al., 2012). Therefore, although the species were formed before LGM, the current altitude range of the four species might still have been influenced by the extreme climate in LGM.

### Differences Between Genetic Structure and Taxonomic Boundaries

External morphological differences and similar environmental niche among *Diabelia* populations in different regions are fascinating. Based on the phylogenetic relationships inferred using chloroplast loci and the result of STRUCTURE analysis (nSSR, Figure 3), only *D. serrata* was found to be a monophyletic group, and consistent with proposed taxonomic boundaries (Landrein and Farjon, 2019). The phylogenetic definition of all other species is at odds with taxonomic boundaries proposed based on morphology alone. There may be several reasons for this: Firstly, the phylogenetic tree used only two chloroplast fragments, with limited mutation sites, and the resulting phylogenetic gene trees may not reflect an accurate species tree for the genus. A second possibility is that populations have diverged, but retained ancestral polymorphisms shared by wider taxa (Sakaguchi et al., 2017), obscuring monophyletic species relationships in the phylogenetic trees. Thirdly, environmentally driven genetic adaptations associated with specific ecotypes may be directly linked to phenotypic plasticity, which can obscure taxonomic boundaries based on morphological traits. Genetic-based adaptive evolution plays a decisive role in expressed morphological traits (Hirano et al., 2017; Sakaguchi et al., 2017). Therefore, the morphological traits expressed in *Diabelia* populations may be primarily based on selective amplification of genetically-determined traits in different geographical habitats. In addition, co-occurring *Diabelia* species can readily hybridize, and we also found a large number of gene infiltration events between species in our analyses of nSSR structure (Figure 3). In the actual sample observation process, we found that hybridization may produce intermediate morphological traits that differ from their parents (*D. serrata* sepal range from 1 to 4; *D. sanguinea* has many flower colors such as red, pink, or dark-pink; Landrein and Farjon, 2019). This may also be one of the reasons for variation of individual morphological characters within a species. We have speculated that *D. ionostachya* var. *tetrasepala* (calyx with five sepals and the adaxial sepal is reduced in size) results from hybridization between *D. serrata* (calyx with 2–3 sepals) and *D. spathulata* (calyx with five sepals). In results of nSSR structure, *D. ionostachya* var. *tetrasepala* individuals from R4, R5, gene infiltration is low (Figure 3). So it is impossible to accurately determine that *D. ionostachya* var. *tetrasepala* is the result of hybridization between *D. serrata* and *D. spathulata*. Intermediate individuals produced by interspecific hybridization do make it difficult to classify species accurately in the field. Additional data may enable the underlying discordance between taxonomic concepts and our molecular data to be correctly interpreted.

## Conclusions

This study presents the first comprehensive analyses of the phylogeny and biogeography of taxa within *Diabelia* using phylogeographic data. Furthermore, the role of the ECS in speciation and species migration was explored with *Diabelia* as a case study. Combining chloroplast, nSSR with ecological niche modeling, we assessed phylogeny and population dynamics in *Diabelia*. Based on our results, a distinct population of *D. ionostachya* was identified in the Yamagata prefecture of northern Japan. The divergence of *Diabelia* species dates back to the mid to late Tertiary. *Diabelia* presents a typical multi-origin pattern of species evolution. *Diabelia* species in China represent primitive populations and the isolating function of the East China Sea in *Diabelia* speciation remains unresolved.

## Data Availability

The datasets generated for this study can be found in Tables S1–S4.

## Author Contributions

H-FW, SL, and SS conceived the ideas and designed methodology. SS and MM collected the materials. K-KZ, HL, and W-XM constructed the species phylogeny cladogram. K-KZ, W-XM, Z-XZ, and TY analyzed the data. H-FW, SL, and RB led the writing of the manuscript. All authors contributed critically to the drafts and gave final approval for publication. All authors contribute equally to the work.

### Conflict of Interest Statement

The authors declare that the research was conducted in the absence of any commercial or financial relationships that could be construed as a potential conflict of interest.

## Supplementary Material

Figure S1 Maximum Likelihood tree (RAxML) for 402 individuals of in *Diabelia* based on concatenated sequences of chloroplast DNA (*trnH-psbA, rpl*32*- trnL*) with seven species (*Kolkwitzia amabilis*_ BOP012293; *Abelia chinensis*_BOP012223; *Dipelta yunnanensis*_BOP012201; *Linnaea borealis*_ BOP012344; *Vesalea floribunda*_BOP022783; *Zabelia buddleioides*_BOP012222; *Zabelia dielsii*_BOP012228) as outgroups.

Figure S2 Principal coordinate analysis (PCoA) and structural analysis of 59 populations of *Diabelia* based on nSSR data using codominant genetic distances.

Figure S3 Structural analysis (nSSR) of 549 individuals based on 69 populations of *Diabelia*. **(a)** ΔK statistics calculated; **(b)** Plot of mean posterior probability values of each K.

Figure S4 Comparison of potential distributions as probability of occurrence for *Diabelia serrata, D. spathulata, D. sanguinea*, and *D. ionostachya* (Landrein and Farjon, 2019), at present and at two climatic scenarios (MIROC, MPI) of the Last Glacial Maximum (LGM, ca. 21,000 years BP). The maximum training sensitivity plus specificity logistic threshold has been used to discriminate between suitable (cutline 0.1–1 areas) and unsuitable habitat. The darker color indicates a higher probability of occurrence.

Figure S5 Population coordinate maps for Ecological Niche Modeling (ENM) analysis of each species (*Diabelia*).

Table S1 GenBank Information for voucher specimens for DNA sequencing (*rpl*32-*trn*L and *trn*H-*psb*A).

Table S2 Longitudinal and latitudinal information of *Diabelia* sampling sites.

Table S3 Probabilities from tests (Wilcoxon' s) for mutation drift equilibrium in 40 populations of *Diabelia* under the infinite allele model (IAM), stepwise mutation model (SMM) and two-phase model (TPM) for nSSR data.

Table S4 The bottleneck effect data analyses of nSSR data.
