# Supplementary figures and images for: Phylogeographic Analysis and Genetic Structure of an Endemic Sino-Japanese Disjunctive Genus Diabelia (Caprifoliaceae)
Source: Front Plant Sci. 2019 Jul 16;10:913. doi: 10.3389/fpls.2019.00913 (PMC6646888; doi:10.3389/fpls.2019.00913)

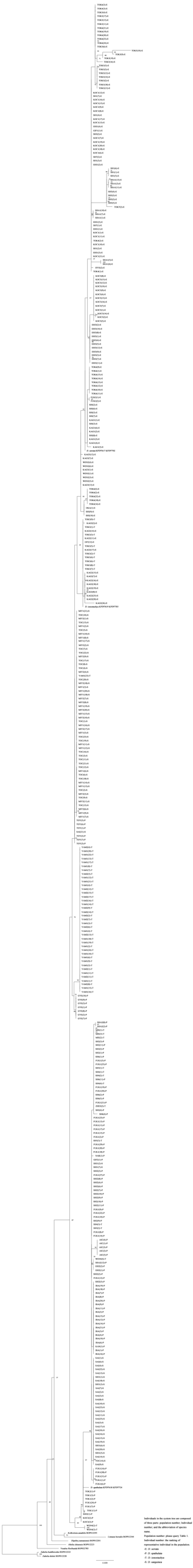

Supplement: Figure S1 — Maximum Likelihood tree (RAxML) for 402 individuals of in Diabelia based on concatenated sequences of chloroplast DNA (trnH-psbA, rpl32- trnL) with seven species (Kolkwitzia amabilis_ BOP012293; Abelia chinensis_BOP012223; Dipelta yunnanensis_BOP012201; Linnaea borealis_ BOP012344; Vesalea floribunda_BOP022783; Zabelia buddleioides_BOP012222; Zabelia dielsii_BOP012228) as outgroups. [file Image_1.JPEG]

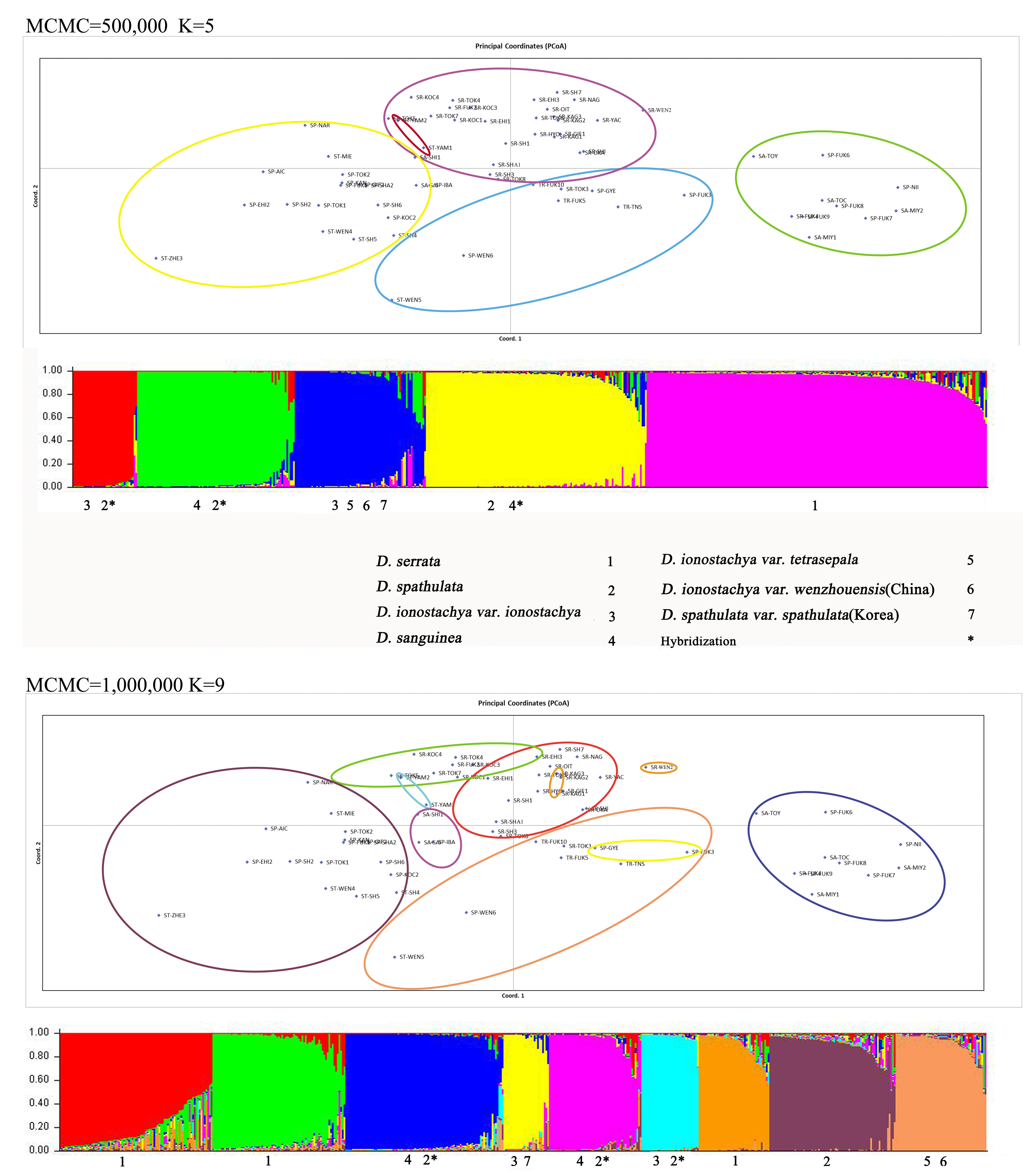

Supplement: Figure S2 — Principal coordinate analysis (PCoA) and structural analysis of 59 populations of Diabelia based on nSSR data using codominant genetic distances. [file Image_2.JPEG]

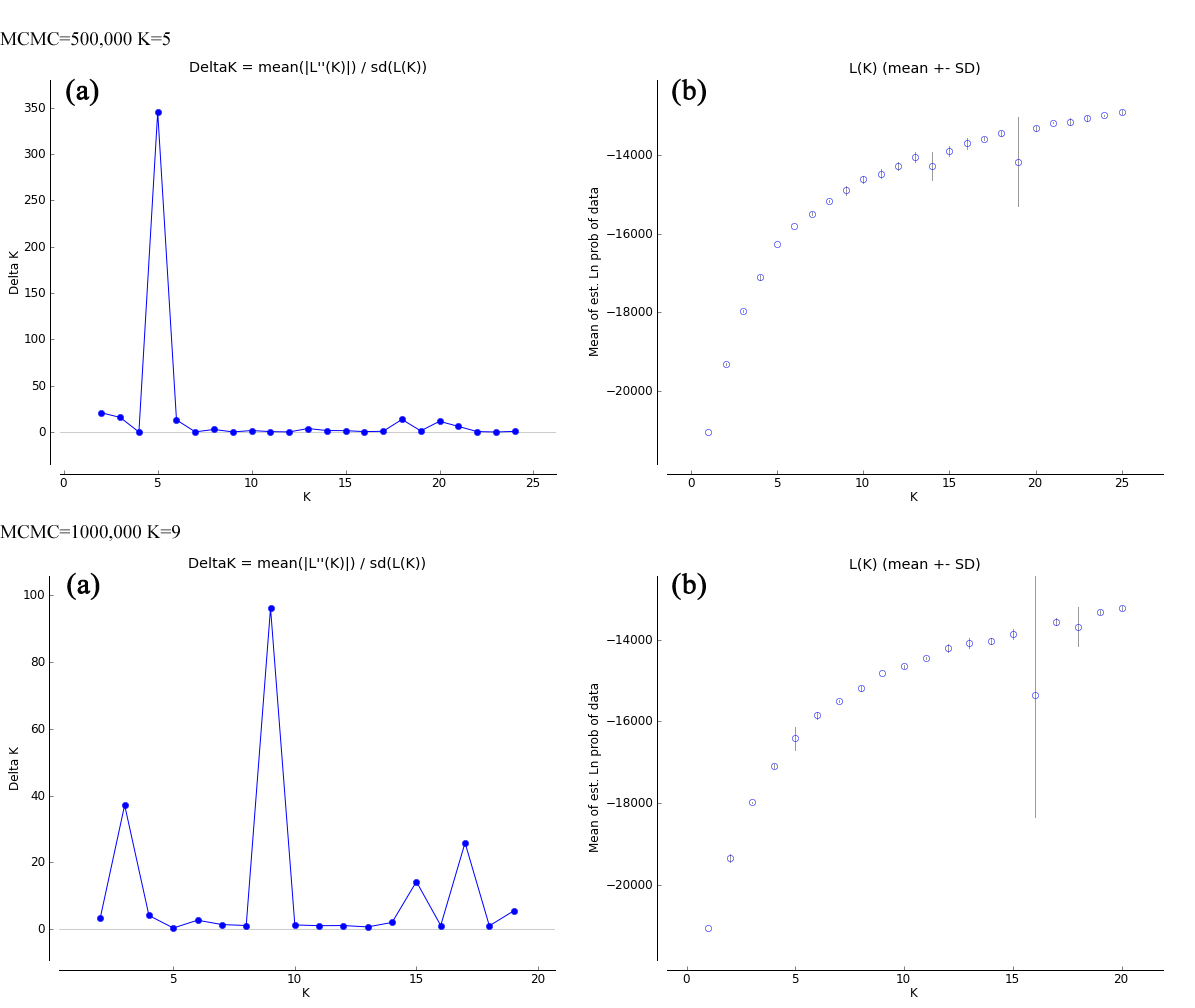

Supplement: Figure S3 — Structural analysis (nSSR) of 549 individuals based on 69 populations of Diabelia. (a) ΔK statistics calculated; (b) Plot of mean posterior probability values of each K. [file Image_3.JPEG]

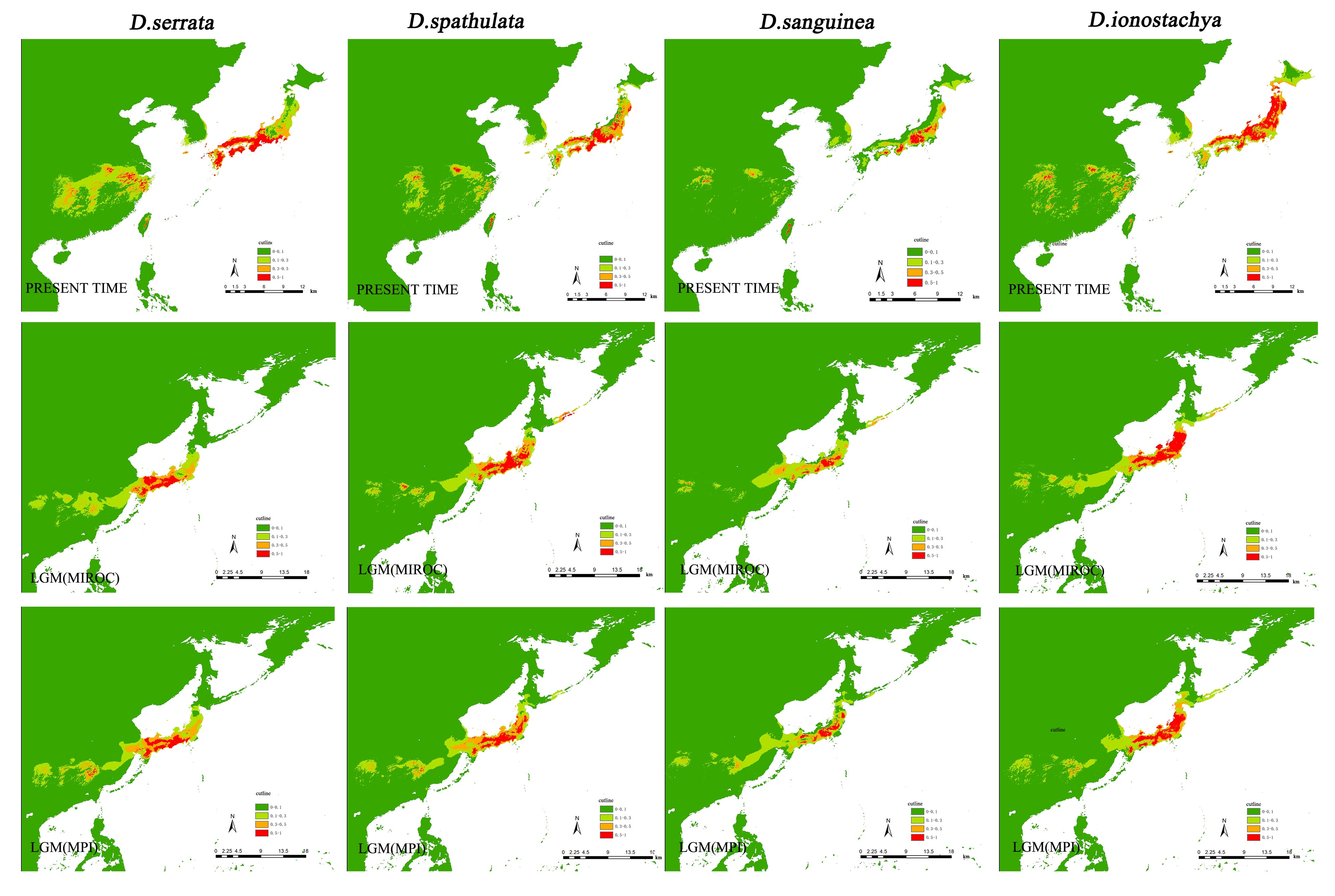

Supplement: Figure S4 — Comparison of potential distributions as probability of occurrence for Diabelia serrata, D. spathulata, D. sanguinea, and D. ionostachya (Landrein and Farjon, 2019), at present and at two climatic scenarios (MIROC, MPI) of the Last Glacial Maximum (LGM, ca. 21,000 years BP). The maximum training sensitivity plus specificity logistic threshold has been used to discriminate between suitable (cutline 0.1–1 areas) and unsuitable habitat. The darker color indicates a higher probability of occurrence. [file Image_4.JPEG]

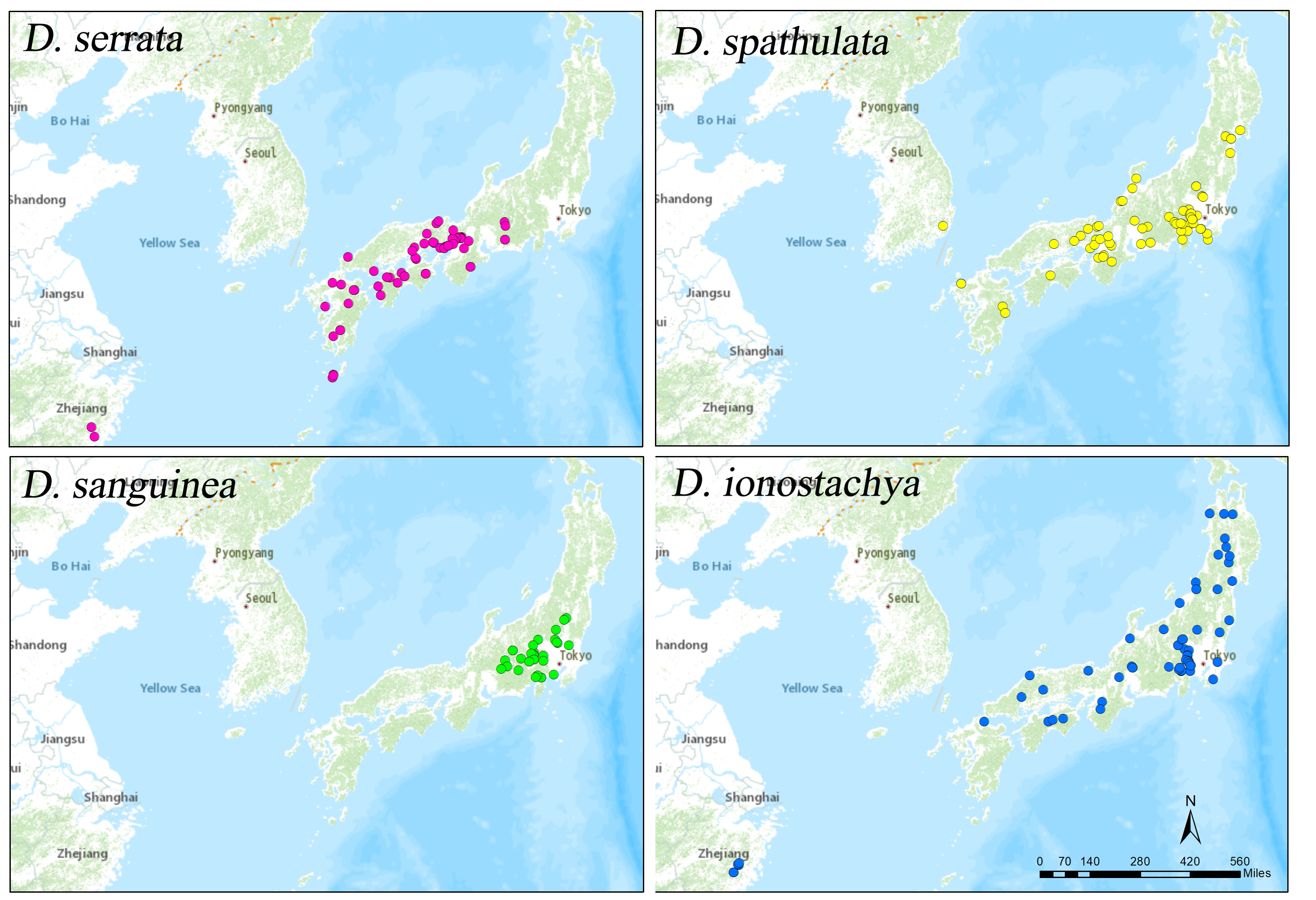

Supplement: Figure S5 — Population coordinate maps for Ecological Niche Modeling (ENM) analysis of each species (Diabelia). [file Image_5.JPEG]
